# Concern for severe COVID-19 disease during the initial two years of pandemic in a rural South African community: A population-based study

**DOI:** 10.1371/journal.pone.0319720

**Published:** 2025-11-20

**Authors:** Armstrong Dzomba, Palesa Mataboge, Nicole K. Kelly, Kelechi Elizabeth Oladimeji, Carren Ginsburg, Bianca Moffett, Chodziwadziwa Kabudula, Kimberley Gutu, Audrey Pettifor, Francesc Xavier Gómez-Olivé, Kathleen Kahn

**Affiliations:** 1 School of Nursing, Psychotherapy and Community Health, Dublin City University, Glasnevin Campus, Dublin, Ireland; 2 School of Built Environment and Development Studies, University of KwaZulu-Natal, Durban, KwaZulu-Natal, South Africa; 3 Department of Epidemiology, University of North Carolina, Gillings School of Global Public Health, Chapel Hill, North Carolina, United States of America; 4 Ezintsha, Faculty of Health Sciences, University of the Witwatersrand, Johannesburg, South Africa; 5 College of Graduate Studies, University of South Africa, Johannesburg, Gauteng, South Africa; 6 Medical Research Council/Wits Rural Public Health and Health Transitions Research Unit (Agincourt), School of Public Health, Faculty of Health Sciences, University of the Witwatersrand, Johannesburg, Gauteng, South Africa; 7 South African Medical Research Council Vaccines and Infectious Diseases Analytics Research Unit, Faculty of Health Sciences, University of the Witwatersrand, Johannesburg, Gauteng, South Africa; University of California Davis School of Medicine, UNITED STATES OF AMERICA

## Abstract

**Background:**

Globally, as of December 2023, over 700 million cases of COVID-19 were confirmed since the initial outbreak late in 2019, claiming around 7 million lives and fuelling widespread fear and anxiety. However, prospective patient-data are unavailable to assess individuals’ perceptions of risk of severe COVID-19 illness, which may imply actual disease severity and inform risk perception for future epidemics.

**Methods:**

We surveyed 1701 adults about their concern for severe COVID-19 disease using a population-based survey of rural South Africans. The initial telephonic survey was conducted between August-October 2020 with a follow-up survey conducted between August-October 2021. Multinomial logistic regression was used to investigate predictors of perceived COVID-19 illness severity (low, medium, or high) controlling for measured confounders.

**Results:**

The prevalence of concern for COVID-19 illness severity in 2020 was 28.7% low, 26.8% moderate and 44.5% severe, with corresponding levels in 2021 of 42%, 31.8% and 29.2%, respectively. Although older age was associated with a lower odds of COVID-19 concern [50–59 years (aOR=0.54, 95% CI: 0.38–0.75)], [≥60 years (aOR=0.41,95% CI: 0.41–0.57)], adults having ≥1 chronic conditions (aOR=1.38,1.00–1.89) or residing outside of the study community (aOR=1.29,1.01–1.65) were more likely to experience moderate and high concern for illness severity, respectively.

**Conclusions:**

Understanding presumptive COVID-19 disease severity may help disentangle various underlying mechanisms behind personal risk assessment. This may inform current thinking and practice of public health emergency medicine in managing emerging and re-emerging respiratory diseases with pandemic potential such as hPMV.

## Background

As of December 2023, over 700 million COVID-19 cases and approximately 7 million deaths were registered globally [1], having generated considerable public anxiety about susceptibility to severe illness over the period leading up to this. The proliferated dissemination of real-time data and metrics on COVID-19 disease impact [2] in hotspot global settings helped shape public opinion often against progression of localized epidemics.

Perception of COVID-19 disease severity is of particular concern in South Africa, country recording the largest COVID-19 epidemic in sub-Saharan Africa, with weekly cases peaking at about 115 845 (June-July 2021) and 5 000 reported deaths (January 2021) [3,4]. By mid-year of 2021, life expectancy declined to match 2006 levels, i.e., height of the HIV epidemic and related mortality [5]. Earlier in 2020, reports of a new South African variant, SARS-CoV-2 (501Y-V2) [6], raised widespread concerns about possible progression, severity of infection, antibody response and vaccine efficacy [7]. Furthermore, early national COVID-19 pandemic preparedness and responses characterized by nationwide restrictions of human mobility (i.e., hard lockdowns), and delayed commencement of vaccine roll-out campaigns may have amplified perceptions of COVID-19 risk. Conversely in 2021, widespread availability of vaccines accompanying the gradual relaxation of COVID-19 non-pharmaceutical interventions (NPIs e.g., mask mandates, social distancing, restricted public gatherings) [8,9], may have waned perception for severe COVID-19 disease. The rapid change in the epidemic trajectory amid the slower public health responses both in South Africa and elsewhere created a void filled by COVID-19 misinformation and disinformation [10] on growing multimedia platforms disseminating often unproven and competing narratives about the origin of the virus, transmission routes, prevention, disease severity and disease management [11–13]. Whether via popular new media or mainstream channels, exposure to sensational and an ever-shifting COVID-19 evidence landscape [14], helped to frame their attitudes and behavior about the fatality of the disease.

Individual self-efficacy and autonomous adoption of preventive behavior are hypothesized to be linked to beliefs about COVID-19 outcomes elsewhere [15–18]. In South Africa, self-perception of COVID-19 risk in the wider population has been investigated [19] with older age and higher education linked with higher risk. Although, predictor data exist locally and globally [20–28] is largely misses measures clinically milder cases as surveillance of a newly emerged pathogen was typically biased towards detecting clinically severe cases [23]. Data detailing transitions of individual concern for COVID-19 disease outcomes during and post-COVID-19 initial outbreak are lacking, although being a vital source of insight for future public health intelligence for emerging and re-emerging acute respiratory infectious threats with pandemic potential and vaccination campaigns [29–31] such as hMPV which is currently resurging in China. A perception-based approach to understand COVID-19 disease severity risk provides an important nuance to measurement given that a clinical perspective and standard criteria for determining endpoints (asymptomatic, mild, and severe cases) dominates current literature [2,32–35]. Further, an emerging priority and key feature of future outbreak control is monitoring public perception and early identification [36] of associated factors particularly among at-risk populations in settings with limited pandemic preparedness infrastructure such as South Africa.

In the current study, we examined the perceptive COVID-19 illness severity continuum and associated demographic, health, and behavioral variables using prospective patient data from a longitudinal population-based survey in the Agincourt Health and Demographic Surveillance System site (AHDSS), in Mpumalanga Province, South Africa. In doing so, we aim to help develop tools to improve the quality of risk analysis and assessment, including timeliness and effectiveness of the national response to acute public health threats.

## Materials and methods

### Study setting

**Fig 1:**
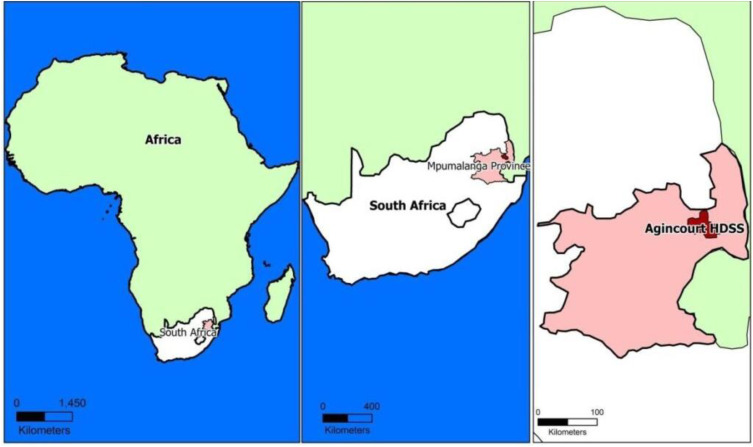
Agincourt health and demographic surveillance system study area.

Data for this study arose from the “Socio-Behavioral-Economic impacts of COVID-19 in rural South Africa” study (SBE study, hereafter). The SBE study is a population-based, representative, randomly sampled cohort of adults 18 years and older selected from the 2019 survey round off the longstanding Agincourt Health and Demographic Surveillance System (HDSS) run by the MRC/Wits Agincourt Research Unit of the University of the Witwatersrand, Johannesburg, South Africa. The larger study frame, Agincourt community, in Mpumalanga Province, constitutes a population research site established in 1992 [37]. The Agincourt HDSS (Fig 1) collects routinely updated detailed information on vital events (births, deaths, and migrations), household composition, measures of household socio-economic status and geo-location of dwellings [37]. Overall, the study focusses on examining both immediate and long-term impacts of COVID-19 lockdown regulations and tracking impacts of individual, household, and structural factors on adherence to prevention guidelines and risk of infection described in previous work [38]. The Human Subject Research Committee (Medical) of University of the Witwatersrand approved an amendment to the Agincourt HDSS ethics clearance of October 2019 with register number M190305, to nest the SBE survey into the Agincourt HDSS data collection protocol. Additional ethics approval for data collection and linkage was provided by the Mpumalanga Department of Health Ethics Committee.

### Design

An initial telephonic survey was conducted between 10 August and 30 October of 2020 (i.e., 6-months since declaration of the national state of disaster and initial enforcement of COVID-19 lockdown regulations), and a follow-up telephonic survey was conducted between August and October 2021, after the public sector rollout of the COVID-19 vaccine in the Province of Mpumalanga in north-east South Africa. Telephone numbers for the sampled participants were obtained from the Agincourt HDSS individual roster, which collates and updates mobile telephone numbers/information for adults in all households. A maximum of three attempts were made to contact sample participants, failing which, due to failed calls, refusal, non-response etc., no further pursuits to recruit those individuals were made without replacement. Given the minimal risk status of this study, verbal consent was obtained, and the Principal Investigator (PI) submitted a phone “script” to the relevant ethics boards, with the former containing a detailed outline of the informed consent. Consent was documented in the Research Assistants’ notes and captured in the RedCap master dataset output collating data analyzed for this study. Each telephonic interview lasted approximately 30-minutes. The questionnaire was translated into xi-Tsonga (the local language) and back translated into English. Interviews were conducted in xi-Tsonga by trained local research assistants and call-centre agents. The survey included questions on; COVID-19 infection, knowledge about COVID-19, risk perception, compliance to prevention, healthcare, and social support, with modules on vaccine uptake introduced in 2021.

### Study participants

**Fig 2:**
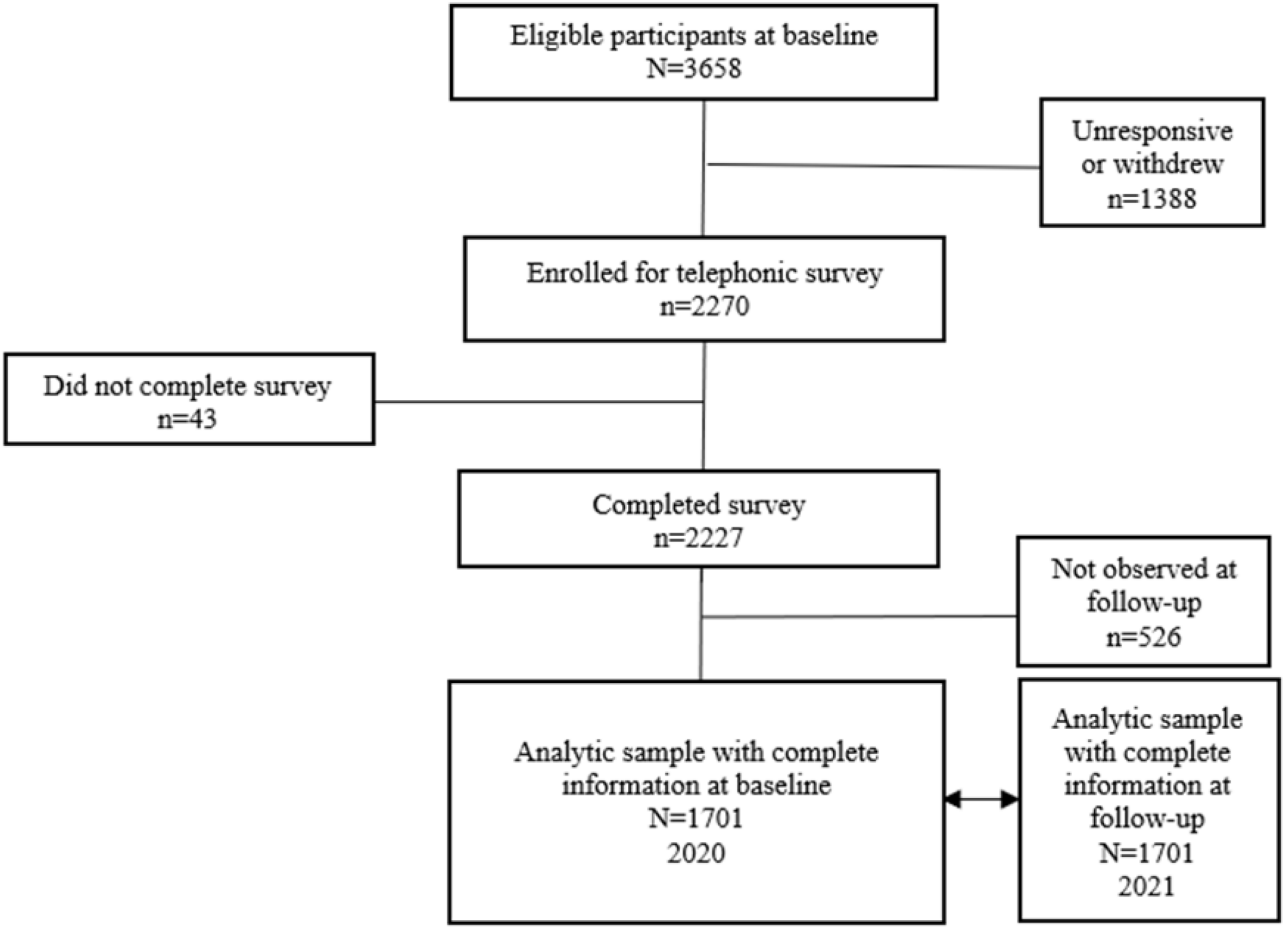
Flowchart for study participants selection.

Fig 2 shows the process flow used to select participants for inclusion in the current study based on the interview call outcomes. The AHDSS hosts over 120 000 residents and ~20 000 households, from whom, we randomly selected participants with aim of recruiting a gender-balanced sample of 2000 individuals, aged 18 years and older. At baseline, 3658 adults were eligible for participation in the SBE study via telephone, of whom 1388 (38%) were excluded for non-response, failed contact or withdrew voluntarily from study, thus 2270 were included in the study. Further exclusions involved 43 who did not complete the survey and 526 who did not participate in the follow-up round in 2021, resulting in a sample size of 1701. In total, 1701 out of the initial 2270 participants who were included in the baseline, were retained in the follow-up. However, the sample was unbalanced by gender as more women (66%) compared to men (34%) were positively responsive to the initial study enrolment invitation at baseline.

### Measures

#### Outcome.

Concern for severe COVID-19 illness was measured at both time points based on the survey question “How concerned are you that you will become seriously ill from COVID-in the next 3-months” with its interval response scale ranging from 1 (not at all) to 4 (extremely). Beyond measuring only clinically severe cases [23] and align with the National Institutes of Health (NIH) criteria for categorising COVID-19 clinical patient status (asymptomatic; mild/moderate; and severe/critical) [30], we recoded responses to the initial survey question to a composite ordinal scale, where 1 = (low), 2 and 3 = (moderate) and 4 = (high).

#### Socio-demographic, behavioral and health covariates.

Covariate factors included sex (male; female), age group (18–29; 30–39; 40–49; 50–59; ≥ 60 years) – time invariant (age and sex), employment status (employed; unemployed-including students, pensioners), and current residency (within HDSS outside HDSS). The total count of chronic conditions were calculated per person and categorised into groups with 0, 1 and 2 or more diseases, from the list of the nine; [(i) HIV, (ii) Tuberculosis (TB), (iii) Hypertension (iv), Diabetes, (v) chronic kidney disease, (vi) Cancer, (vii) cardiovascular disease, (viii) Asthma + other lung diseases (ix), Mental health condition].

We measured compliance to COVID-19 protocols by averaging across six items which assessed the self-reported level of ease to adhere to a selection of universal NPIs for COVID-19 infection mitigation at home or in public spaces. Participants were asked six questions on how easy it was to: (i) wash their hands with soap and water for 20 seconds, (ii) clean surfaces in their houses regularly, (iii) wear a mask when in public places, (iv) keep distance of 1.5 to 2 metres from other people at all times when out of the house, (v) stay in a separate room in the house to other household members when sick, (vi) stay at home except to get food or essential medication. These questions established in-built temporal order in that they required participants to provide responses based on preventive behaviour from 6 months prior to data collection. We summed Likert response values of these set of questions (where the possible range was 5–16) with a lower and higher sum of scores representing increased and lower level of compliance, respectively. We then recoded these sums to scale (Cronbach’s α = 0.77) and constructed compliance to COVID-19 protocols categories, where 11/16 = low, 10 = medium and 5/9 = high compliance.

### Statistical analysis

**Fig 3:**
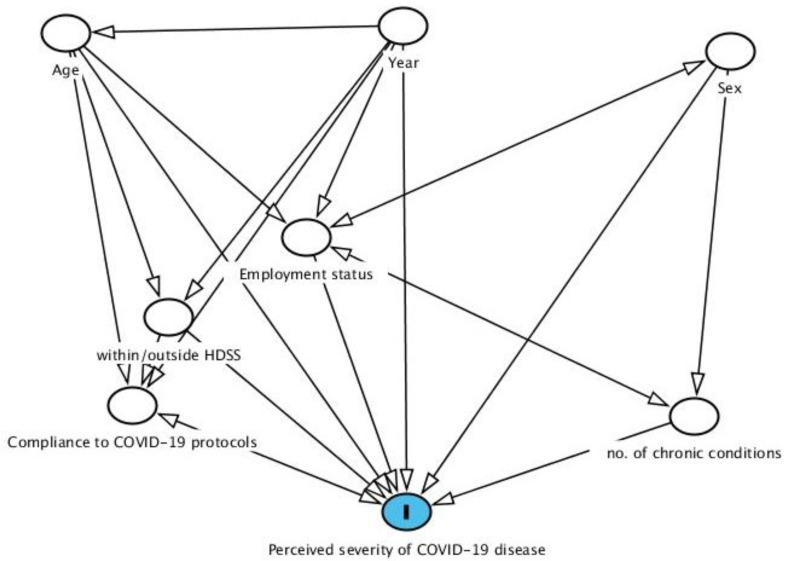
Directed acyclic graph illustrating the relationships between the exposures of interest and the primary outcome: Perceived risk of severe COVID-19 illness.

**Fig 4:**
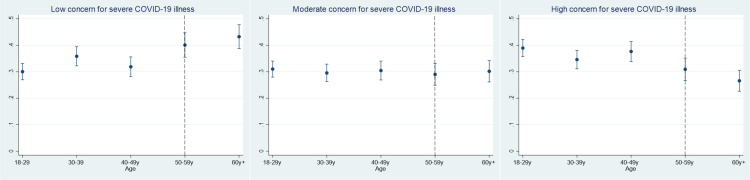
Marginal predicted proportions for age category in relation to level of COVID-19 illness severity concern.

**Fig 5:**
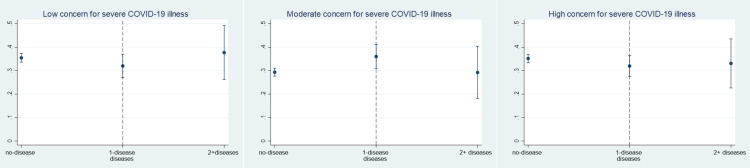
Marginal predicted proportions for no. of chronic conditions in relation to level of COVID-19 illness severity concern.

Five sets of analyses were performed to explore levels of concern for severe COVID-19 illness and examine associated risk factors. First, we summarised the participants’ socio-behavioural and health factors at baseline employing descriptive and inferential analysis (frequencies, proportions, and measures of association) stratified by sex to examine nuances given our unbalanced sample. Second, we identified socio-behavioural and health predictors of concern for severe COVID-19 illness, using standard Pearson’s chi-square (χ2) tests for each of the two survey rounds. Third, we then analysed associations with perceived COVID-19 illness severity using multinomial logistic regression models with random effects (for time). Fourth, predicted probabilities were calculated to illustrate predicted risks for each outcome category with respect to two predictors (age and number of chronic conditions (Figs 4 and 5). Our statistical analysis was guided by a theoretical model to identify and limit potentially confounding variables that could bias the estimates of perceived COVID-19 illness severity as depicted in a directed acyclic graph (DAG) (Fig 3), designed in the software DAGgity 2.2. DAGs, or causal diagrams, are commonly used in epidemiologic analyses to graphically represent causal and potentially biasing pathways (i.e., confounders). For this analysis, variables included in the DAG were identified from the literature and from the authors’ existing substantive subject knowledge. Further, the use of DAGs to inform these analyses ensures a rigorous and causally driven approach, which is desirable to avoid overfitted multivariable modelling which often results in confounding or unclear interpretations of the effect estimates (i.e., Table 2 fallacy) [39]. Therefore, we present results from DAG-driven models, with hypothesis graphically represented in Fig 3. All statistical analyses were two-sided (alpha = 0.05), and all analyses were conducted using STATA 17 (Stata Statistical Software: Release 17. College Station, TX: StataCorp LP. StataCorp. 2022).

To estimate the total, unbiased effect of several different socio-demographic-behavioral-health characteristics on perceived severity of COVID-19 illness, we adjusted for measured confounders in separate multivariable models. Our predictors of interest included: age, calendar year, employment status, residency within/outside HDSS, number of chronic conditions, sex and level of compliance to COVID-19 protocols was necessary. The relationships between these variables are displayed in Fig 3. We hypothesized a direct causal association between all the variables and the outcome signified by arrows pointing towards variable (**I**) in Fig 3. For example, an arrow from the number of chronic conditions to perceived severity of COVID-19 illness represents our expectation that a change in number of chronic conditions causes a change in perceived severity of COVID-19 disease. Whereas other variables have both direct and indirect links with the outcome, for instance survey year linked with the outcome in linear fashion, alternatively, it is connected to the outcome via employment status, implying the latter’s mediatory role.

## Results

### Baseline socio-demographic-behavioral and health characteristics

Table 1 presents baseline socio-demographic-behavioral and health characteristics of the cohort (N = 1701), with median age of 37.5 years [interquartile range (IQR): 27.1–51.4]. Most participants were women (n = 1088, 63.8%). Overall, approximately a quarter of participants reported being employed, 19.2% resided outside the study area and 27.8% had ≥ 1 chronic condition.

**Table 1:** Baseline characteristics of a cohort of adults living in rural South Africa, August – October 2020 (N = 1701).

	Overall N = 1 701		Female N = 1 088 (63.8%)		Male N = 613 (36.2%)		Statistics
	N	%	n	%	n	%	
Age category							
18-29y	599	32.9	307	28.3	251	41.1	χ2(4) = 40.64, p = 0.000**
30-39y	372	21.9	246	22.7	125	20.4	
40-49y	299	17.6	205	18.9	93	15.2	
50-59y	219	12.9	156	14.4	64	10.5	
≥ 60y	212	14.7	174	15.7	80	12.8	
Employment status							
Employed	420	24.7	216	19.9	202	33.1	χ2(1) = 48.59, p = 0.000**
Unemployed	1 281	75.3	872	80.1	411	66.9	
Current residence							
Within HDSS	1 374	80.8	883	81.2	472	80.3	*χ2(1) = 0.24, p = 0.619*
Outside HDSS	327	19.2	205	18.8	121	19.7	
No. of chronic conditions							
None	1 228	72.2	722	66.4	505	82.4	χ2(2) = 73.65, p = 0.000**
1-condition	406	23.9	305	28.1	101	16.6	
2 or more conditions	77	3.9	61	5.5	7	1.1	
Compliance to COVID-19 NPIs							
Low	365	21.5	212	19.5	153	25.1	χ2(2) = 9.44, p = 0.009*
Intermediate	544	32.0	356	32.8	186	30.5	
High	792	46.5	520	47.7	274	44.4	

Degrees of freedom indicated in parentheses, level of significance *p < 0.05, **p < 0.01

Regarding preventive behavior, nearly half of the cohort (n = 792, 46.5%) reported high levels of compliance to COVID-19 NPIs, whereas two thirds (n = 544, 32.0%) reported moderate/intermediate compliance and 21.5% (n = 365) reported low compliance. The prevalence of these characteristics differed significantly by sex, with 33.1% of men vs 19.9% of women being employed, 16.6% of men vs 33.6% of women having at least one chronic condition and 25.1% of men vs 19.5% of women having low compliance to COVID-19 NPIs.

### Sociodemographic, behavioral and health predictors of concern for severe COVID-19 illness

Based on our classification of the outcome, 28.7% of participants had low, 26.8% moderate and 44.5% high concern for severe COVID-19 illness in 2020. One year later, the corresponding estimates were 42%, 31.8% and 29.2%, respectively. The socio-demographic, behavioral and health characteristics and potential predictors of concern for severe COVID-19 illness in 2020 and 2021 are provided in Table 2. Consistently across all covariates, high concern for severe COVID-19 illness in 2021 was nearly half of 2020 levels. In contrast, moderate concern for COVID-19 significantly increased slightly over the same period for individuals intermediately and highly compliant with COVID-19 protocols.

**Table 2:** Socio-demographic covariates of concern for severe COVID-19 illness among a sample of adults living in rural South Africa (N = 1701).

	2020				2021			
	Low, n (%)	Moderate, n (%)	High, n (%)		Low, n (%)	Moderate, n (%)	High, n (%)	
Sex								
Male	232 (28.8)	208 (25.8)	366 (45.4)	*χ2 (2) = 4.63, p = 0.099*	261 (45.6)	181 (31.6)	131 (22.9)	*χ2 (2) = 0.71, p = 0.70*
Female	411 (28.7)	392 (27.4)	630 (43.9)		443 (40.2)	372 (33.8)	287 (26.0)	
Age categories								
18-29y	189 (25.6)	194 (26.3)	354 (48.1)	*χ2 (8) = 16.39, p < 0.05*	166 (35.6)	161 (34.6)	139 (29.8)	*χ2 (2) = 29.85, p < 0.001*
30-39y	148 (30.2)	134 (27.4)	208 (42.5)		147 (40.0)	120 (32.5)	101 (27.5)	
40-49y	99 (25.6)	112 (28.4)	184 (46.6)		135 (41.2)	107 (32.6)	86 (26.2)	
50-59y	95 (32.9)	71 (25.6)	123 (42.5)		111 (47.0)	81 (34.3)	44 (18.7)	
60y+	112 (34.2)	89 (27.1)	127 (38.7)		144 (52.2)	84 (30.4)	48 (17.4)	
Employment status								
Employed	467 (27.7)	451 (26.8)	767 (45.5)	*χ2 (2) = 4.03, p = 0.13*	485 (42.2)	382 (33.6)	282 (24.5)	*χ2 (2) = 0.34, p = 0.85*
Unemployed	176 (31.8)	149 (26.9)	229 (41.3)		219 (41.6)	171 (32.5)	136 (25.9)	
Current Residency								
Within DSA	587 (32.4)	437 (24.1)	787 (43.5)	*χ2 (2) = 72.44, p < 0.001*	542 (41.5)	425 (32.5)	339 (26.0)	*χ2 (2) = 3.18, p = 0.204*
Outside DSA	56 (13.1)	163 (38.1)	209 (48.8)		162 (43.9)	128 (34.7)	79 (21.4)	
No. of chronic conditions								
None	463 (28.8)	406 (25.3)	739 (45.9)	*χ2 (4) = 12.74, p < 0.05*	518 (42.9)	390 (32.4)	297 (24.7)	*χ2 (2) = 2.92, p = 0.571*
1-condition	146 (26.9)	171 (31.6)	225 (41.5)		159 (38.7)	143 (34.8)	109 (26.5)	
2 or more conditions	34 (38.2)	23 (25.8)	32 (36.0)		26 (44.8)	20 (34.5)	12 (20.7)	
Compliance to COVID-19 NPIs								
Low	102 (27.4)	110 (29.6)	160 (43.0)	*χ2 (4) = 89.54, p < 0.001*	93 (36.1)	87 (33.9)	77 (30.0)	*χ2 (4) = 20.71, p < 0.001*
Intermediate	220 (41.2)	134 (25.1)	180 (33.7)		162 (36.3)	175 (39.2)	109 (24.5)	
High	168 (20.9)	221 (27.5)	416 (51.6)		449 (46.2)	291 (29.9)	232 (23.9)	

Row percentages in parentheses, () after χ2 is degrees of freedom. NPIs = non-pharmaceutical interventions.

In 2020, those who were highly compliant with COVID-19 NPIs were more likely to be highly concerned about severe COVID-19 (51.6%, Table 2). Those residing outside of the HDSS had the least prevalence of low concern for COVID-19 (13.1%). In 2021, these levels decreased and increased, respectively. Further, in 2021 individuals aged 18–29 years and ≥60 years had the lowest (35.6%) and highest percentage (52.2%) of individuals with low severe COVID-19 concern. In total 911 of 1701 (54% of the sample population) had received the COVID-19 vaccine by wave 2 data collection in 2021.

### Random-effects multinomial logistic regression models

Estimates for longitudinal changes related to the levels of concern for severe COVID-19 illness are presented in Table 3 below. Covariate categories of age, residency, compliance with COVID-19 protocols and calendar time were independently associated with either mild and/or high concern compared to low concern for severe COVID-19 illness. Those aged 50−59 years (OR = 0.70; 95% CI: 0.53–0.91) and ≥60 years (OR=0.68; 95% CI: 0.53–0.88) and calendar year 2021 (OR=0.82; 95%CI: 0.69–0.9)7 were found to have reduced odds of moderate concern. A lower odds of high concern for severe COVID-19 disease was associated with age categories 30−39 years (OR=0.72; 95%CI: 0.57–0.91), 50−59 years (OR=0.57; 95% CI: 0.43–0.74), ≥ 60 years (OR=0.45; 95%CI: 0.34–0.58), moderate adherence to COVID-19 protocols (OR=0.63; 95%CI: 0.49–0.82) and year 2021 (OR=0.36;95%CI: 0.30–0.42).

**Table 3:** Unadjusted estimates from random-effects multinomial logistic regression models of concern for COVID-19 illness severity (1701).

Variables	Moderate			High		
	SE	OR	95% CIs	SE	OR	95% CIs
Sex: [Male]						
Female	0.10	0.13	0.95–1.35	0.10	1.08	0.91–1.29
Age category: [18-29y]						
30-39y	0.10	0.80	0.63–1.03	**0.09**	0.72*	**0.57–0.91**
40-49y	0.12	0.92	0.71–1.18	0.11	0.87	0.68–1.12
50-59y	**0.10**	0.70**	**0.53–0.91**	**0.08**	0.57**	**0.43–0.74**
≥ 60y	**0.09**	0.68**	**0.53–0.88**	**0.06**	0.45**	**0.34–0.58**
Employment status: [Unemployed]						
Employed	0.09	0.92	0.76–1.11	0.08	0.85	0.71–1.03
Chronic conditions: [None]						
1-condition	0.11	1.08	0.89–1.31	0.09	0.95	0.78–1.16
2-or more conditions	0.20	0.95	0.63–1.43	0.17	0.79	0.47–1.81
Current residency: [Within DSA]						
Outside DSA	**0.17**	1.59**	**1.29–1.95**	0.13	1.21	0.98–1.50
Compliance to COVID-19 protocols: [Low]						
Moderate	0.10	0.82	0.63–1.03	**0.08**	0.63**	**0.49–0.82**
High	0.10	0.81	0.65–1.04	0.10	0.88	0.70–1.10
Year: [2020]						
2021	**0.07**	0.82**	**0.69–0.97**	**0.03**	0.36**	**0.30–0.42**

OR=Odds Ratio; SE = Standard Error, 95% CI = 95% Confidence Intervals, DSA = Demographic Surveillance Area, *p < 0.05, **p < 0.01, employment status, chronic conditions, current residency, compliance to COVID-19 protocols were time-variant.

Several socio-demographic and behavioral characteristics were significantly associated with moderate compared to low concern for severe COVID-19 illness (Table 4). Odds ratios (OR) for those aged 50–59 years (OR=0.66; 95% CI: 0.48–0.90) and ≥60 years (OR=0.62; 95% CI: 0.46–0.84) compared to individuals aged 18–29 years indicated lower concern for severe COVID-19 illness. Similar diminished concern was found for 2021: OR=0.80; 95% CI: 0.65–0.98 compared to 2020. However, living outside the study area was associated with higher odds ratios (OR=1.65;95% CI: 1.31–2.07) compared to living in the study area. Among those with one chronic condition, the odds of being moderately concerned for COVID-19 disease was 1.3 times that compared to those without any chronic health conditions (OR=1.38; 95% CI: 1.00–1.89). The average probability of being intermediately concerned of a serious COVID-19 threat was 0.36 in the presence of one underlying health condition, whereas this probability was 0.31 in the absence of any health condition (Fig 4). Thus, individuals had a higher chance of having moderate concern for severe COVID-19 if they had one health challenge as shown in Fig 4 and Table 4.

**Table 4:** Estimates from random-effects multinomial logistic regression models of concern for COVID-19 illness severity outcome (1701).

Variables	Moderate			High		
	SE	aOR	95% CI	SE	aOR	95% CIs
Sex: [Male]						
Female	0.11	1.20	0.98–1.47	0.13	1.18	0.95–1.47
Age category: [18-29y]						
30-39y	0.10	0.77	0.58–1.01	0.10	0.70*	**0.53–0.93**
40-49y	0.12	0.91	0.68–1.22	0.14	0.89	0.66–1.21
50-59y	**0.10**	0.66**	**0.48–0.90**	0.09	0.54**	**0.38–0.75**
≥ 60y	**0.09**	0.62**	**0.46–0.84**	0.07	0.41**	**0.29–0.57**
Employment status: [Unemployed]						
Employed	0.09	0.94	0.76–1.16	0.11	0.93	0.74–1.16
Chronic conditions: [None]						
1-condition	0.13	1.38*	**1.00–1.89**	0.17	1.01	0.74–1.40
2-or more conditions	0.25	0.92	0.47–1.81	0.29	0.86	0.44–1.67
Current residency: [Within DSA]						
Outside DSA	0.17	1.65**	**1.31–2.07**	0.16	1.29*	**1.01–1.65**
Compliance to COVID-19 protocols: [Low]						
Moderate	0.11	0.84	0.64–1.10	0.11	0.72*	**0.54–0.96**
High	0.11	0.91	0.71–1.18	0.16	1.16	0.89–1.51
Year: [2020]						
2021	**0.81**	0.80*	**0.65–0.98**	0.03	0.32**	**0.26–0.40**

aOR=Adjusted Odds Ratio; SE = Standard Error, 95% CI = 95% Confidence Intervals, DSA = Demographic Surveillance Area, *p < 0.05, **p < 0.01, employment status, chronic conditions, current residency, compliance to COVID-19 protocols were time-variant.

The strength and direction of associations for moderate concern of severe COVID-19 illness was consistent with the high concern outcome class, with only minor differences observed across covariate categories (Table 4). Our regression results suggest that effect is significantly different in those aged 30–39 years (aOR=0.70; 95% CI: 0.53–0.93), those aged 50–59 years (aOR=0.54; 95% CI: 0.38–0.75) and those aged ≥60 years (aOR=0.41; 95% CI: 0.41–0.57) compared with those 18–29 years, as illustrated in Fig 5 and Table 4. Across the two study waves, the association with living outside versus within the study area held for higher concern for COVID-19 illness (aOR=1.29; 95% CI: 1.01–1.65). In comparison to 2020, the 2021 odds of perceived highly severe COVID-19 illness were 68% lower. We also found that self-reported moderate compliance to NPIs predicted a negative association with higher concern for severe COVID-19 disease: aOR=0.72; 95% CI: 0.54–0.96.

## Discussion

The purpose of this study was to examine the spectrum of concern for COVID-19 illness severity and identify associated risk factors over the initial two years of the pandemic among individuals from rural Mpumalanga Province, South Africa. Our results demonstrate a shift from higher levels of concern early in the pandemic when little was known about the virus (August-October 2020) towards moderate and lower levels of concern one year later (August-October 2021). Moreover in 2021, in South Africa as was elsewhere [8,10,14], collective minimization of severity coupled with ‘pandemic fatigue’ may have simultaneously lowered concern for COVID-19. This change in public perception in combination with increased knowledge of COVID-19 may have shifted concern for severe COVID-19 illness by the second study round. Additionally, 56% of our sample had received a COVID-19 vaccine by the 2021 data collection wave, plausibly contributing to this positive outlook by protecting participants from the threat of severe disease and death.

COVID-19 perceived severity data is rare distinctly for rural or underserved communities in South Africa [8,40] with exceptions in sub-Saharan Africa [41]. Several of our findings are consistent with those from previous work. We note similarities of our 2020 findings against a contemporary study recruiting an outpatient sample of caregivers attending a children Measles and Rubella (MR) vaccination program at outreach sites in Zambia [42] which generally mirrors the healthcare delivery levels in rural South Africa. In the Zambian MR vaccination study, the proportion of those who considered COVID-19 a severe disease was consistent with our study (44% vs. 43%). Notably results were comparable, despite employing different outcome variable definitions, group vs. personal severity, with the MR study based on an ecological level in contrast to our individual level in terms of unit of analysis. Elevated COVID-19 severity observed in the Zambian MR vaccination study is potentially a function of selection bias [43], with a homogenous sample of women caregiver participants (as was possibly the case in that study) being more likely to be sensitive to potential danger to children in their care [44]. Similarly, our study recruited both women (64%) and men (36%), and even with an overrepresentation of women, sex did not account for increased levels of COVID-19 concern. We thus echo others’ [8] suggestions that correlates of self-perception of pandemic risk are themselves endogenous often producing conflicting results if not acknowledged – a feature of studies during the pandemic.

Older age was associated with lower levels of concern for severe COVID-19 illness, which may be illustrative of lower risk literacy common among older adults in rural settings. Evidence from a nationally representative survey in South Africa found significantly increased risk perception among the educated and older population groups [8], as the epidemic unfolded in 2021. However, our results potentially demonstrate the backward linkage between education (as a proxy for health literacy) and age in a typical rural community in sub-Saharan Africa. First, education and literacy rates for the elderly vary across life stages in South Africa, and the study area has one of the largest groups of persons above 60 years with no schooling [45]. In keeping with this theory, young adults 30–39 years had lower severe COVID-19 concern (Table 4) corresponding with their greater public health knowledge and awareness of the less severe disease outcomes given their innate age-health advantage compared to those who are older. Alternatively, it is possible to suggest that the cohort of older adults who lived through the devastation of early HIV epidemic in South Africa had honed COVID-19 coping skills and resilience due to experience of serious adversity to their physical and mental health [46]. They may also hold a strong sense of community and social cohesion which is a potential source of general positive outlook despite facing an ‘existential’ COVID-19 threat [47–49].

Heightened concern for severe disease risk was linked to residence beyond the home environment, likely reflecting the possible different COVID-19 awareness compliance levels in diverse contexts. Those located in urban areas during the study area typically reside in high population density settings under poor living arrangements. Such areas often have dilapidated sanitation and water infrastructure and informal tightly spaced, low-cost and shared housing settlements which hugely undermined compliance efforts to COVID-19 NPIs (e.g., social distancing and handwashing) [50]. A cohort study of young adult migrants and residents from the same Agincourt study area found that continuous migrants (a large proportion of which were based in Gauteng during this period of the pandemic) were more inclined to employ protection measures (such as mask wearing and hand washing) compared to rural-based cohort participants [51]. This suggests contextual variation in COVID-19 impact and behaviors/beliefs potentially highlighting differences between urban and rural respondents over this time as demonstrated elsewhere [52]. The SARS-CoV-2 seroprevalence in urban settings appeared to be greater than in rural areas [53] with spatial epidemiology and the varying degree of exposure are potential explanations for this heterogeneity.

Further, we found that moderate compliance to COVID-19 protocols was associated with lower odds of concern for severe COVID-19 symptoms. However, it is difficult to establish whether mild adherence to prevention behavior precedes presumptive optimistic COVID-19 outcomes. During the inter-survey period in South Africa, general pandemic fatigue may have confounded this exposure-outcome relationship given the prolonging epidemic [54] and authorities’ relaxation of NPIs [55].

Despite being a singular target of the national COVID-19 communication, prevention and vaccination effort, patients with one pre-existing chronic condition held no better self-perceived health status. By 2021, it was official knowledge that underlying conditions were associated with a higher risk of severe COVID-19 [56]. The Agincourt site is an emerging high chronic disease prevalence setting [57], with HIV, hypertension and obesity driving multimorbidity (≥2 co-existing health conditions). Over half of those reporting one chronic condition are living with HIV, which may have influenced their disease expectations for COVID-19. The innate hazard for progression to severe illness and prolonged clinical recovery time are well-established outcomes for immunosuppressed patients, such as people living with HIV [30]. COVID-19 beliefs for individuals living with HIV may hinge upon prior experience of HIV-related stigma and social distancing [58,59], fueling fear of COVID-19-related discrimination, which is known to impact motivations for COVID-19 testing and vaccination uptake [60,61].

This study contained many strengths, including providing a necessary focus on understanding the evolution of presumptive COVID-19 disease severity and elucidating the underlying characteristics behind personal risk assessment. Furthermore, we used data from a large, representative study nested within the longstanding Agincourt HDSS during two crucial time-points of the pandemic in South Africa. Despite this study’s strengths, there are three key limitations. First, the study may be subject to temporal bias in which it is possible that conceptual aspects of illness concern and COVID-19 behavior shifted in tandem with the rapidly unfolding epidemic and universal response landscape between the early pandemic in 2020 and 2021 when disease severity may have been interpreted differently and compliance to COVID-19 was no longer obligatory. An inherent limitation of phone surveys (and interview technic for this study) is susceptibility to coverage bias which diminishes the representativeness of the interviewed sample [62]. Unfortunately, class adjustments weights were not available to rigorously counteract the sample skewness by gender for our study results to reflect the population representativeness more accurately. We performed analyses stratified by sex given suggestions that women are prone to distress about COVID-19 compared to men although there was no a-priori reason to suggest that differences in rates of illness differ between the sexes. Thirdly, we used measures of chronic disease status based on self-report, with no clinical diagnoses and confirmatory information available.

Our study implies the need to ongoingly adapt public health risk communication in tandem with public perception, which plays an ever-increasing role in voluntary uptake of preventive behavior in the era of COVID-19 and beyond. The reduced risk perception for those beyond age 50 years may be illustrative of lower risk literacy or learned striving against adversity, which has implications for ongoing risky COVID-19 behavior/beliefs. We reiterate others’ recommendations that effectively disseminating health-related risk information will require customizing interventions to the needs of specific groups such as older adults [63]. To do this, multi-media should be carefully deployed in managing expectations including encouraging adherence to containment measures [64]. For example, misperceptions related to COVID-19 were mostly driven by social media videos allegedly showing congested hospital emergency departments in different world regions [65]. During an emerging outbreak (such as the N1H1 swine-flu epidemic in Europe, 2009) there may be uncertainties about infectiousness and case fatality of the disease. Beyond only communicating with the public about ‘what is known’ (the certainties), health authorities should also communicate ‘what is not known’ (the uncertainties) [66] to ensure that people hold an objective view of the pandemic, a buffer to COVID-19 misinformation campaigns [67]. Other group-targeted campaigns should be personalized, such as leveraging technology for the youth and those mobile (e.g., social media) or intermediaries (such as schoolteachers, popular community figures) [68]. Public health experts also need to collaborate with communities to better understand their specific challenges and needs during unparalleled crises. In South Africa, reference exists for social cohesion, mobilizing individuals, families, and communities to support public health responses through the unique experience with the HIV epidemic. Novel and dynamic ways to foster individual agency [69] are needed to maintain long-term public compliance with protective measures during the COVID-19 and future crises.

## Conclusion

Our study analyzed the continuum of concern for COVID-19 infection and its associated factors in a rural community in South Africa with limited health and sanitation infrastructure from 2020 through 2021. Elevated individual concern for severe disease in 2021 declined to nearly half of 2020 levels likely in response to the ambivalent population-level pandemic fatigue, easing of NPIs and expanded vaccine rollout program by the national government and availability of the COVID-19 vaccine. Overall, age, residence, chronic disease status, and level of compliance to COVID-19 explained varying levels of concern for COVID illness severity over time. Public health intelligence practitioners should collaborate more with communities to understand unique challenges and needs in future crises mitigation planning. It is an emerging consensus that public health messaging should pivot to monitoring public perception and early identification of protective factors for COVID-19 and related epidemics, particularly for vulnerable populations with limited health and sanitation infrastructure, such as rural settings in sub-Saharan Africa.

## Supporting information

S1 Data SBE study dataset. (ZIP)

## Abbreviations

WHO: World Health Organization
NICD: National Institutes of Communicable Diseases
STATSSA: Statistics South Africa
HIV: Human Immune Virus
ICU: Intensive Care Unit
NIH: National Institutes of Health
COPD: Chronic Obstructive Pulmonary Disease
AHDSS: Agincourt Health and Demographic Surveillance System
SBE: Socio-Behavioral-Economic impacts of COVID-19
TB: Tuberculosis
DAG: Directed Acyclic Graphs
NPIs: Non-Pharmaceutical Interventions
DSA: Demographic Surveillance Area
